# Supplementary material for: Managing minor ailments and pharmacy services: How do people make their decisions?
Source: PLoS One. 2025 Aug 26;20(8):e0330706. doi: 10.1371/journal.pone.0330706 (PMC12380283; doi:10.1371/journal.pone.0330706)
Supplement: S1 File — (S1 File.PDF) [file pone.0330706.s001.pdf]

S1 File. Questionnaire "Exploration of the opinions in the management of minor ailments"

Foreword:

Hello,

In the context of my master thesis in pharmaceutical sciences, I carry out an exploratory opinion study in the population living in the French speaking part of Switzerland (≥18 years old) in order to explore the criteria influencing the decision making when dealing with minor ailments that affect you, decrease your capacities and/or your quality of life (for example: sore throat, cough, cystitis). The management of these symptoms depend on your decision (do nothing, self-medication, pharmaceutical/medical/emergency consultation).

The goal is to explore your needs and your expectations to improve the functioning of the healthcare system.

10 to 15 minutes are needed to complete the following questionnaire.

By answering to the questionnaire, you agree that the data collected are stored in the internal server of Unisanté and analyzed by the research team of the Pharmacy of Unisanté, in a deidentified manner, in accordance to the data protection law. You also enable the data to be published in a scientific journal or in other research projects lead by the research team of Unisanté.

In advance, I thank you for your time, your participation is precious.

In case of questions or interest related to the project, don't hesitate to contact me: […]

Part 1: Preferences

Through three fictive scenarios, we aim to explore the criteria that you take in account when dealing with minor ailments.

Rank these three scenarios from the most to least serious:

a. *Since a week, you have a stuffy nose, a dry cough and 39.5°C fever.*
b. *Since 24 hours, you had 6 diarrhea episodes with moderate pain linked to cramps and a loss of appetite. Your general condition is enhancing slowly but the symptoms remain.*
c. *Since 5 days, you find red fresh blood in your feces after going to the toilets and on the paper when you wipe off your feces. You don't have any other symptom.*

Choice:

1. Most serious scenario
2. Neutral scenario
3. Less serious scenario

For the scenario a. Since a week, you have a stuffy nose, a dry cough and 39.5°C fever.

Rank in order of preference (1. First choice, 2. Second choice (if 1. isn't available) and 3. Third choice (if the 1. And 2. aren't available) the three preferred shares amongst the following:

1. Go to an emergency service
2. Consult a GP
3. Call a telephonic center of on-call GP
4. Call a telephonic center operated by an insurance company
5. Go to a pharmacy
6. Self-medication
7. Do nothing
8. Don't know
9. Other: please specify

For the scenario b. Since 24 hours, you had 6 diarrhea episodes with moderate pain linked to cramps and a loss of appetite. Your general condition is enhancing slowly but the symptoms remain. Rank in order of preference (1. First choice, 2. Second choice (if 1. isn't available) and 3. Third choice (if the 1. And 2. aren't available) the three preferred shares amongst the following:

1. Go to an emergency service
2. Consult a GP
3. Call a telephonic center of on-call GP
4. Call a telephonic center related to an insurance company
5. Go to the pharmacy
6. Self-medication
7. Do nothing
8. Don't know
9. Other: please specify

For the scenario c. Since 5 days, you find red fresh blood in your feces after going to the toilets and on the paper when you wipe off your feces. You don't have any other symptom.

Rank in order of preference (1. First choice, 2. Second choice (if 1. isn't available) and 3. Third choice (if the 1. And 2. aren't available) the three preferred shares amongst the following:

1. Go to an emergency service
2. Consult a GP
3. Call a telephonic center of on-call GP
4. Call a telephonic center related to an insurance company
5. Go to the pharmacy
6. Self-medication
7. Do nothing
8. Don't know
9. Other: please specify

When responding to these three scenarios, how did you take decisions? Indicate the criteria that you integrated in your reflexion (Very important, important, neutral, little importance, no importance)

1. Geographical proximity (distance) with the care provider
2. Time to deal with symptoms
3. Costs
4. Health insurance model
5. Symptom severity
6. Opening hours
7. Access to medicine
8. Don't know
9. Don't want to answer
10. Other criteria (yes/no)
    If yes – specify

If you don't know how to handle minor ailments, who do you contact? Multiple answers possible.

- I ask my relatives
- I go to the pharmacy
- I call a telephonic centrale of on-call GP
- I call a telephonic center operated by an insurance company
- I go to the GP
- I go to the emergencies
- I consult Internet
- Other
- Don't know

Indicate the minor ailments that would make you visit a pharmacy (yes I go to the pharmacy for a consultation or a counselling, yes I go to the pharmacy to buy some medicines without prescriptions, no I don't go to the pharmacy if I have these symptoms, unapplicable, I don't know, I don't want to answer.

Burn

Conjunctivitis

Constipation

Asthma crisis

Erectile dysfunction

Eczema

Urinary tract infection

Back pain

Head pain

Tick bite

Cutaneous mycosis (feet, hands)

Vaginal mycosis

Allergic rhinitis

We suggest you a list of criteria, tell us which ones make you consult in pharmacies if you must deal with minor ailments.

Geographical proximity

Reference pharmacy (a pharmacy where you always go, where your file is known)

Opening hours

Waiting time

Counter service

Consultation room

Quality of service

Staff skills

Available services (for example: vaccination, diabetes screening, opioid rotation, blood pressure measurement)

Symptom severity

Access to medicine

Health insurance coverage

Other: please specify

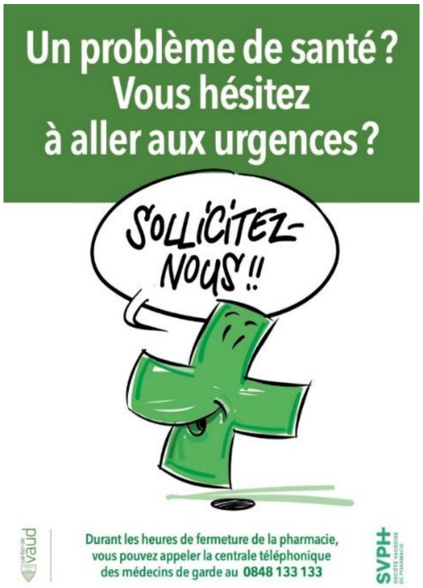

Have you ever seen this flyer (or a similar flyer)?

1. Yes
2. No
3. Don’t want to answer

Since few years, new alternative insurance models have emerged and restrain insured to use pharmacies or telemedicine centers as first point of entry of the health system before going to a GP. The pharmacies where insured are oriented are partner pharmacies of health insurance compagnies.

In exchange, the insured choosing these models benefit of a reduction of monthly premiums (amount that is paid to the insurance and that differ depending on the chosen deductible) and of the coverage of certain pharmacy services.

It is the case for Sympany and it’s models casamed pharm, OKK and its model Casamed select, Groupe mutuel and its model Primaflex and SWICA and its model MEDPHARM.

Do you know this type of insurance model?

1. Yes
2. No
3. Don’t want to answer

Is their function clear?

1. Yes
2. No
3. Don’t know

Would you be ready to subscribe to an alternative insurance model that orient you in a pharmacy?

1. Yes
2. No
3. Don’t know
4. Don’t want to answer

Are reduced premiums a sufficient incentive to subscribe to this alternative insurance model?

1. Yes
2. No
3. Don’t know
4. Don’t want to answer

Are there any other criteria that influence you in your decision?

1. Yes
2. No
3. Don’t want to answer

If yes – please specify

A growing number of minor ailments can be managed in pharmacies. Netcare is a pharmaceutical prestation in certified pharmacies that enables the management of minor ailments. The consultations also offers a teleconsultation with a general practitioner.

Did you know Netcare before its description?

1. Yes
2. No
3. Don’t want to answer

Would you be ready to use Netcare?

1. Yes
2. No
3. Don’t know
4. Don’t want to answer

Right now, certain alternative insurance models reimburse the Netcare consultation. It is the case for certain alternative insurance models OKK, Sympany or Swica.

Knowing that a Netcare consultation can be taxed up from 30-40 chf (without the medicines), would you be ready to pay the price of the service out-of-your-pocket if the service was not reimbursed.

1. Yes
2. No

3. Don’t know
4. Don’t want to answer

If no – How much would you agree to pay? (price in chf)

Soignez-moi.ch is an online telemedicine platform that collaborates with 180 pharmacies. The patient can go in a partner pharmacy and benefit from a consultation to manage the minor ailment or respond to an online questionnaire. A GP calls the patient afterwards to confirm the procedure. In both cases, a prescription can be delivered if needed. The price of this service is reimbursed by the all of the insurance models, same as the complementary tests that can be made in the pharmacy in the context of a soignez-moi.ch service (blood sugar level, angina screening)

Were you aware of the existence of Soignez-moi.ch?

1. Yes
2. No
3. Don’t want to answer

Would you use Soignez-moi.ch?

1. Yes
2. No
3. Don’t know
4. Don’t want to answer

Since 2019, the pharmacists can deliver medicines without having a medical prescription in order to manage minor ailments. These medicines were only available with a medical prescription before 2019.

Were you aware of the possibility to get these prescription based medicines in pharmacies without requiring a medical prescription?

1. Yes
2. No
3. Don’t want to answer

Would you be ready to go to a pharmacy when you have minor ailments in order to receive medicines under prescription from your pharmacist?

1. Yes
2. No
3. Don’t know
4. Don’t want to answer

Right now, the cost of the deliverance of under prescription medicines by the pharmacist is not reimbursed by the insurance

Knowing that the pharmacist can ask for 15 to 20 francs (without the cost of the medicine) for the consultation needed to deliver a medicine that is usually available under a prescription, would you be ready to pay the service out of your pocket?

1. Yes
2. No
3. Don't know
4. Don't want to answer

If no - how much would you agree to pay?

Part 2: Sociodemographic data

How do you identify?

1. Male
2. Female
3. Other
4. Don't know
5. Don't want to answer

What is your age range?

1. 18-30 years
2. 31-50 years
3. 51-64 years
4. 65-80 years
5. >80 years
6. Don't want to answer

What is your highest educational level?

1. Compulsory schooling
2. Secondary vocational
3. High school diploma
4. Professional diploma
5. University, higher education
6. Unknown Other
7. Don't know
8. Don't want to answer

Do you work in the healthcare field?

1. Yes
2. Non
3. Don't want to answer

If yes – select the corresponding domain

1. Pharmacy field
2. Insurances
3. Other healthcare profession
4. Don’t want to answer

What is your residency place?

1. Appenzell Inner-Rhodes
2. Appenzell Outer-Rhodes
3. Argovia
4. Basle-City
5. Basle-Coutry
6. Bern
7. Freiburg
8. Geneva
9. Glarus
10. Grisons
11. Jura
12. Lucerne
13. Neuchâtel
14. Nidwald
15. Obwald
16. Schaffhouse
17. Schwyz
18. Soleure
19. St. Gall
20. Thurgovia
21. Ticino
22. Uri
23. Vaud
24. Valais
25. Zug
26. Zurich

What is your insurance type :

1. Basic mandatory insurance
2. Alternative model
3. Don’t have a swiss insurance
4. Other
5. Don’t know
6. Don’t want to respond

If alternative model – please specify

1. GP model
2. HMO model
3. Telemedicine model
4. Mixed-model with pharmacies
5. Other: please specify

What is your deductible amount? (Your deductible is the yearly amount chosen that the insured needs to pay out of the pocket before the insurance reimburses the costs)

1. 300 chf
2. 500 chf
3. 1000 chf
4. 1500 chf
5. 2000 chf
6. 2500 chf
7. Other: please specify
8. Don’t know
9. Don’t want to respond

Do you have a referring physician?

1. Yes
2. No
3. Don’t know
4. Don’t want to answer

Do you have a referring pharmacy?

1. Yes I have one
2. I have multiple ones
3. No I always change
4. No I don’t need one
5. Don’t know
6. Don’t want to answer

How would you qualify your health status?

1. Excellent
2. Very-good
3. Good
4. Unsatisfying
5. Poor
6. Don’t know
7. Don’t want to answer

Do you have a chronic illness?

1. Yes
2. No
3. I have multiple ones
4. Don’t know
5. Don’t want to answer

Thank you for taking the time to complete the questionnaire!

If you have any comments or suggestions, you can leave them bellow: ____________
