# Supplementary material for: Managing minor ailments and pharmacy services: How do people make their decisions?
Source: PLoS One. 2025 Aug 26;20(8):e0330706. doi: 10.1371/journal.pone.0330706 (PMC12380283; doi:10.1371/journal.pone.0330706)
Supplement: S1 Table — (S1 Table.DOCX) [file pone.0330706.s002.docx]

**Supporting informations**

**S1 Table. Examples of pharmacy services to manage minor ailments available in Switzerland**

| **Services (year)** | **Description** | **Accessibility** | **Cost** | **Funding** |
| --- | --- | --- | --- | --- |
| **Autonomous prescribing (2019)** (1) | Provision of in-depth advice on a health problem following advanced triage, usually given in a consultation room (13). The service was introduced in 2019 following the revision of the Therapeutic Products Act enabling pharmacists to autonomously prescribe over 100 previously “prescription-only medicines” (14) | Can be delivered to everyone (sometimes with paediatric age limits) in all pharmacies according to commercial strategy, but with a lack of national implementation (15) | Fixed by the community pharmacy (≈ 20 CHF + the cost of medicines) (2) | Patient |
| **Prescribing via structured prescribing arrangement** “**Netcare”**  **(2012) (3)** | Triage service including a structured assessment of the symptoms, urgent red flags warranting immediate referral, suggested triage outcomes and treatment recommendations according to decision trees. Triage outcomes could be under the pharmacist responsibility (e.g. guided self-medication, self-care, prescription medicine without physician validation, referrals) or with telemedicine support if needed (12) (16). | Can be delivered to everyone but only in member pharmacies (20%, n=371/1844) (4) | 15 or 48 CHF (with telemedicine support + cost of medicines) (5) | Patient or partial coverage by some insurance models |
| **Prescribing under GP supervision**  **“Soignez-moi.ch”**  **(2023) (6) (7)** | Access to a digital GP platform available directly online for the population or via a partner pharmacy. The pharmacist uses decision trees to assess symptoms and detect red flags. A GP always confirms the decision. | Can be delivered to everyone but only in member pharmacies (10%, n=180/1844) | 59 CHF (excluding medicine costs and prescribed complementary exams) (8) | Coverage by mandatory health insurance |

**References**

1. OFSP, Office fédéral de la santé publique [Internet]. 2023 [cité 3 nov 2023]. Remise simplifiée de médicaments de la liste B. Disponible sur: https://www.bag.admin.ch/bag/fr/home/medizin-und-forschung/heilmittel/abgabe-von-arzneimitteln.html

2. Amara SB. SVPH. 2022 [cité 18 janv 2024]. Délivrance de médicaments des listes B+/B- : Protocole Galenica. Disponible sur: https://www.svph.ch/download/rich/4475/kfntbr4k.pdf/Primary%20Care%20%20Pr%C3%A9sentation%20Mme%20Ben%20Amara%2031.3.pdf

3. Stämpfli D, Winkler BA, Vilei SB, Burden AM. Assessment of minor health disorders with decision tree-based triage in community pharmacies. Research in Social and Administrative Pharmacy. 2022;18(5):2867‑73.

4. Faits et chiffres 2020: Pharmacies suisses. Berne: Pharmasuisse; 2020 p. 1‑94.

5. Erni P, Von Overbeck J, Reich O, Ruggli M. netCare, a new collaborative primary health care service based in Swiss community pharmacies. Research in Social and Administrative Pharmacy. 2016;12(4):622‑6.

6. Soignez-moi.ch [Internet]. 2024 [cité 3 nov 2023]. Devenir pharmacie partenaire. Disponible sur: https://www.soignez-moi.ch/pharmacies-partenaires

7. Corset JM. 24 heures. 2020 [cité 2 janv 2024]. Les entreprises et le coronavirus – Soignez-moi.ch, la consultation médicale en ligne à portée de clic. Disponible sur: https://www.24heures.ch/soignez-moi-ch-la-consultation-medicale-en-ligne-a-portee-de-clic-516594540277

8. Soignez-moi.ch. Patients. 2023 [cité 3 nov 2023]. Tarifs et remboursement: Remboursement par votre assurance maladie. Disponible sur: https://www.soignez-moi.ch/assurances-maladie

9. ÖKK [Internet]. 2024 [cité 6 déc 2023]. Premier interlocuteur au choix via Telmed, l’appli ou la pharmacie. Disponible sur: https://www.oekk.ch/fr/clients-prives/assurances/assurance-de-base/telmed-appli-pharmacie

10. The NHS Minor Ailment Service at your local pharmacy [Internet]. [cité 6 juin 2024]. Disponible sur: http://www.gov.scot/publications/nhs-minor-ailment-service-local-pharmacy-2/

11. Baqir W, Learoyd T, Sim A, Todd A. Cost analysis of a community pharmacy « minor ailment scheme » across three primary care trusts in the North East of England. Journal of Public Health. 1 déc 2011;33(4):551‑5.

12. Amador-Fernández N, Gastelurrutia MÁ, García-Cárdenas V. Development of self-care in Spanish community pharmacies. Exploratory Research in Clinical and Social Pharmacy. déc 2023;12:100337.

13. NHS England — Midlands » NHS Community Pharmacist Consultation Service (CPCS) [Internet]. [cité 6 juin 2024]. Disponible sur: https://www.england.nhs.uk/midlands/nhs-england-and-nhs-improvement-midlands-work/nhs-community-pharmacist-consultation-service-cpcs/

14. Drug Tariff Part VIC: NHS Community Pharmacist Consultation Service (CPCS) [Internet]. NHS UK; [cité 6 juin 2024]. Disponible sur: https://www.nhsbsa.nhs.uk/sites/default/files/2023-06/Drug%20Tariff%20Part%20VIC%2007062023.pdf

15. NHS England — Midlands » Think pharmacy first [Internet]. [cité 6 juin 2024]. Disponible sur: https://www.england.nhs.uk/midlands/nhs-england-and-nhs-improvement-midlands-work/community-pharmacy-extended-care-services/

16. GOV.UK [Internet]. [cité 6 juin 2024]. Pharmacy First letter to contractors. Disponible sur: https://www.gov.uk/government/publications/pharmacy-first-contractual-framework-2023-to-2025/pharmacy-first-letter-to-contractors

17. NHS Pharmacy First Service [Internet]. [cité 6 juin 2024]. Disponible sur: https://www.bobatpharmacy.co.uk/pages/NHS_Pharmacy_First_Service

18. Dineen-Griffin S, Garcia-Cardenas V, Benrimoj SI. An Australian minor ailments scheme : Evaluation of an integrated approach by community pharmacists and general medical practitioners [Internet]. UNIVERSITY OF TECHNOLOGY SYDNEY; 2019 [cité 6 juin 2024]. Disponible sur: https://www.uts.edu.au/sites/default/files/2019-11/Full%20Report%20%28wl%29.pdf
