# Supplementary material for: Managing minor ailments and pharmacy services: How do people make their decisions?
Source: PLoS One. 2025 Aug 26;20(8):e0330706. doi: 10.1371/journal.pone.0330706 (PMC12380283; doi:10.1371/journal.pone.0330706)
Supplement: S2 Table — (S2 Table.DOCX) [file pone.0330706.s003.docx]

| **Scenarios** | **Perceived severity of the symptoms** | **Type of management preferred** |
| --- | --- | --- |
| **Diarrhea**: In the last 24 hours, you have had 6 episodes of diarrhea with moderate pain associated with cramps and loss of appetite. Your general condition is gradually improving, but the symptoms continue. |  | 1. Self-medication (38%, n=192) 2. Pharmacy (20%,n=103) 3. GP (19%, n=98) 4. Do nothing (13%, n=64) |
| **Dry cough:** For the past week, you have had a blocked nose, a dry cough and a fever of 39.5° |  | 1. GP (39%, n=197) 2. Self-medication (36%,n=185) 3. Pharmacy (14%, n=69) 4. Do nothing (4%, n=20) |
| **Rectal bleeding**: For the past five days, you have been finding fresh red blood in your stools after each trip to the toilet and on the paper when you wipe yourself. You have no other symptoms. |  | 1. GP (58%, n=296) 2. ED (9%, n=45) 3. Telephone helpline (8%, n=39) 4. Pharmacy (7%, n=37) |
