# Supplementary material for: Managing minor ailments and pharmacy services: How do people make their decisions?
Source: PLoS One. 2025 Aug 26;20(8):e0330706. doi: 10.1371/journal.pone.0330706 (PMC12380283; doi:10.1371/journal.pone.0330706)
Supplement: S3 Table — (S3 Table.DOCX) [file pone.0330706.s004.docx]

| “Y*es I go to the pharmacy for a consultation or a counselling*” |  |
| --- | --- |
| *“Yes I go to the pharmacy to buy some medicines without prescriptions”* |  |
