# Supplementary material for: Managing minor ailments and pharmacy services: How do people make their decisions?
Source: PLoS One. 2025 Aug 26;20(8):e0330706. doi: 10.1371/journal.pone.0330706 (PMC12380283; doi:10.1371/journal.pone.0330706)
Supplement: Attachment — Submitted filename: PONE-D-25-05729revised.pdf [file pone.0330706.s006.pdf]

PLOS ONE

Managing minor ailments and pharmacy services: how do people make their decisions?

--Manuscript Draft--

**Manuscript Number:**	PONE-D-25-05729
**Article Type:**	Research Article
**Full Title:**	Managing minor ailments and pharmacy services: how do people make their decisions?
**Short Title:**	Managing minor ailments and pharmacy services: how do people make their decisions?
**Corresponding Author:**	Clemence Perraudin<br>Center for Primary Care and Public Health (Unisanté), University of Lausanne, Switzerland<br>Lausanne, SWITZERLAND
**Keywords:**	Community pharmacy; Minor ailment; symptom management; Decision Making; pharmacy services
**Abstract:**	ABSTRACT<br>Background<br>Healthcare costs and shortages of healthcare professionals are challenges for healthcare systems. Optimal resource allocation is needed, notably in the management of minor ailments. Community pharmacy services (e.g. minor ailment schemes) are often underused by the population. This study aims to explore the decision-making process when people are managing minor ailments: What criteria influence their choices among the management options? What is their level of information and willingness to use and pay for pharmacy services?<br>Methods<br>In this cross-sectional online survey, participants were invited to complete a self-administered questionnaire (convenience sampling) from 07.11.2023 to 08.12.2023. The questionnaire explored general decision-making process in minor ailment management through three clinical scenarios, factors influencing whether to choose pharmacy services to manage minor ailments, as well as public knowledge about three pharmacy services in Switzerland and their willingness to use and pay for them.<br>Results<br>The proportion of valid questionnaires was 99.8% (508/514). Perceived severity of symptoms and time to deal with symptoms were the most important criteria in making the management decision. The more serious the symptoms were perceived, the less the pharmacy and self-medication were privileged. Respondent's decision about whether to go to the pharmacy when dealing with minor ailments depended mainly on the perceived staff's skills and direct access to medicines. Pharmacy services were little known by the public. Respondents were more willing to use autonomous prescribing than other services, with low willingness to pay out of their pocket.<br>Conclusions<br>This study shows that respondents are willing to use pharmacy services to manage minor ailments but are unaware of their existence and have low willingness to pay. Communication and uniformization of pharmacy services are needed, as well as the identification of right incentives to achieve the political goal of adequate orientation in the healthcare system when managing minor ailments.
**Order of Authors:**	Clemence Perraudin
	Léa Charnaux
	Jérôme Berger
**Additional Information:**	
**Question**	**Response**
**Financial Disclosure**	Yes

Powered by Editorial Manager® and ProduXion Manager® from Aries Systems Corporation

Enter a financial disclosure statement that describes the sources of funding for the work included in this submission. Review the submission guidelines for detailed requirements. View published research articles from *PLOS ONE* for specific examples.<br><br>This statement is required for submission and **will appear in the published article** if the submission is accepted. Please make sure it is accurate.<br><br>**Funded studies**<br>Enter a statement with the following details:<br>• Initials of the authors who received each award<br>• Grant numbers awarded to each author<br>• The full name of each funder<br>• URL of each funder website<br>• Did the sponsors or funders play any role in the study design, data collection and analysis, decision to publish, or preparation of the manuscript?<br><br>Did you receive funding for this work?	
Please add funding details.<br>as follow-up to "**Financial Disclosure**<br><br>Enter a financial disclosure statement that describes the sources of funding for the work included in this submission. Review the submission guidelines for detailed requirements. View published research articles from *PLOS ONE* for specific examples.<br><br>This statement is required for submission and **will appear in the published article** if the submission is accepted. Please make sure it is accurate.	All the master’s thesis in pharmacy at the University of Geneva and conducted at Unisanté are financially supported by a regional organisation, the Community Pharmacist Association in Vaud region (SVPh or Société Vaudoise de Pharmacie).

Powered by Editorial Manager® and ProduXion Manager® from Aries Systems Corporation

**Funded studies**<br>Enter a statement with the following details:<br>• Initials of the authors who received each award<br>• Grant numbers awarded to each author<br>• The full name of each funder<br>• URL of each funder website<br>• Did the sponsors or funders play any role in the study design, data collection and analysis, decision to publish, or preparation of the manuscript?<br><br>Did you receive funding for this work?"	
Please select the country of your main research funder (please select carefully as in some cases this is used in fee calculation).<br>as follow-up to "**Financial Disclosure**<br><br>Enter a financial disclosure statement that describes the sources of funding for the work included in this submission. Review the submission guidelines for detailed requirements. View published research articles from *PLOS ONE* for specific examples.<br><br>This statement is required for submission and **will appear in the published article** if the submission is accepted. Please make sure it is accurate.<br><br>**Funded studies**<br>Enter a statement with the following details:<br>• Initials of the authors who received each award<br>• Grant numbers awarded to each author<br>• The full name of each funder<br>• URL of each funder website<br>• Did the sponsors or funders play any role in the study design, data collection and analysis, decision to publish, or preparation of the manuscript?<br><br>Did you receive funding for this work?"	SWITZERLAND - CH
**Competing Interests**	The authors have declared that no competing interests exist.

Use the instructions below to enter a competing interest statement for this submission. On behalf of all authors, disclose any competing interests that could be perceived to bias this work—acknowledging all financial support and any other relevant financial or non-financial competing interests.<br><br>This statement is required for submission and **will appear in the published article** if the submission is accepted. Please make sure it is accurate and that any funding sources listed in your Funding Information later in the submission form are also declared in your Financial Disclosure statement.<br><br>View published research articles from *PLOS ONE* for specific examples.<br><br>**NO authors have competing interests**<br><br>Enter: *The authors have declared that no competing interests exist.*<br><br>**Authors with competing interests**<br><br>Enter competing interest details beginning with this statement:<br><br>*I have read the journal's policy and the authors of this manuscript have the following competing interests: [insert competing interests here]*<br><br>* typeset	
**Ethics Statement**<br><br>Enter an ethics statement for this submission. This statement is required if the study involved:<br><br>• Human participants<br>• Human specimens or tissue<br>• Vertebrate animals or cephalopods<br>• Vertebrate embryos or tissues	N/A ;<br>According to the Swiss Federal Law on Research Involving Human Beings [47], no submission to the Ethics Commission for Research on Human Beings (CER-VD) [48] was required, as the data were de-identified. Tacit consent was obtained during the questionnaire's completion S1 File.

• Field research<br><br>Write "N/A" if the submission does not require an ethics statement.<br><br>General guidance is provided below. Consult the submission guidelines for detailed instructions. **Make sure that all information entered here is included in the Methods section of the manuscript.**	

Powered by Editorial Manager® and ProduXion Manager® from Aries Systems Corporation

**Format for specific study types**<br><br>**Human Subject Research (involving human participants and/or tissue)**<br>• Give the name of the institutional review board or ethics committee that approved the study<br>• Include the approval number and/or a statement indicating approval of this research<br>• Indicate the form of consent obtained (written/oral) or the reason that consent was not obtained (e.g. the data were analyzed anonymously)<br><br>**Animal Research (involving vertebrate animals, embryos or tissues)**<br>• Provide the name of the Institutional Animal Care and Use Committee (IACUC) or other relevant ethics board that reviewed the study protocol, and indicate whether they approved this research or granted a formal waiver of ethical approval<br>• Include an approval number if one was obtained<br>• If the study involved *non-human primates*, add *additional details* about animal welfare and steps taken to ameliorate suffering<br>• If anesthesia, euthanasia, or any kind of animal sacrifice is part of the study, include briefly which substances and/or methods were applied<br><br>**Field Research**<br><br>Include the following details if this study involves the collection of plant, animal, or other materials from a natural setting:<br>• Field permit number<br>• Name of the institution or relevant body that granted permission	
**Data Availability**<br><br>Authors are required to make all data underlying the findings described fully available, without restriction, and from the time of publication. PLOS allows rare exceptions to address legal and ethical concerns. See the PLOS Data Policy and FAQ for detailed information.	No - some restrictions will apply

Powered by Editorial Manager® and ProduXion Manager® from Aries Systems Corporation

A Data Availability Statement describing where the data can be found is required at submission. Your answers to this question constitute the Data Availability Statement and **will be published in the article**, if accepted.<br><br>**Important:** Stating 'data available on request from the author' is not sufficient. If your data are only available upon request, select 'No' for the first question and explain your exceptional situation in the text box.<br><br>Do the authors confirm that all data underlying the findings described in their manuscript are fully available without restriction?	
**Describe where the data may be found in full sentences. If you are copying our sample text, replace any instances of XXX with the appropriate details.**<br><br>• If the data are **held or will be held in a public repository**, include URLs, accession numbers or DOIs. If this information will only be available after acceptance, indicate this by ticking the box below. For example: *All XXX files are available from the XXX database (accession number(s) XXX, XXX.)*.<br>• If the data are all contained **within the manuscript and/or Supporting Information files**, enter the following: *All relevant data are within the manuscript and its Supporting Information files.*<br>• If neither of these applies but you are able to provide **details of access elsewhere**, with or without limitations, please do so. For example:<br><br>*Data cannot be shared publicly because of [XXX]. Data are available from the XXX Institutional Data Access / Ethics Committee (contact via XXX) for researchers who meet the criteria for access to confidential data.*<br><br>*The data underlying the results presented in the study are available from (include the name of the third party*	All data and documentation are available under "restricted access" on the Unisanté data repository for research purposes only. Access request can be made by research teams at : https://doi.org/10.16909/dataset/56

and contact information or URL).<br>• This text is appropriate if the data are owned by a third party and authors do not have permission to share the data.<br><br>* typeset	
Additional data availability information:	

Powered by Editorial Manager® and ProduXion Manager® from Aries Systems Corporation

Click here to access/download;Manuscript;PlosOne_Management of minor

Managing minor ailments and pharmacy services: how do people make their decisions?

Authors: Léa Camille Charnaux[1,2], Jérôme Berger[1,2,3,4] and Clémence Perraudin[1,2,3,4] *

[1] School of Pharmaceutical Sciences, University of Geneva, Geneva, Switzerland

[2] Institute of Pharmaceutical Sciences of Western Switzerland, University of Geneva, University of Lausanne, Lausanne, Switzerland,

[3] Center for Research and Innovation in Clinical Pharmaceutical Sciences, Lausanne University Hospital and University of Lausanne, Lausanne, Switzerland

[4] Department of Ambulatory Care, University Center for Primary Care and Public Health, University of Lausanne, Lausanne, Switzerland

* Correspondence: clemence.perraudin@unisante.ch

Jérôme Berger and Clémence Perraudin are co-last authors of the paper.

ABSTRACT

Background

Healthcare costs and shortages of healthcare professionals are challenges for healthcare systems. Optimal resource allocation is needed, notably in the management of minor ailments. Community pharmacy services (e.g. minor ailment schemes) are often underused by the population. This study aims to explore the decision-making process when people are managing minor ailments: What criteria influence their choices among the management options? What is their level of information and willingness to use and pay for pharmacy services?

Methods

In this cross-sectional online survey, participants were invited to complete a self-administered questionnaire (convenience sampling) from 07.11.2023 to 08.12.2023. The questionnaire explored general decision-making process in minor ailment management through three clinical scenarios, factors influencing whether to choose pharmacy services to manage minor ailments, as well as public knowledge about three pharmacy services in Switzerland and their willingness to use and pay for them.

Results

The proportion of valid questionnaires was 99.8% (508/514). Perceived severity of symptoms and time to deal with symptoms were the most important criteria in making the management decision. The more serious the symptoms were perceived, the less the pharmacy and self-medication were privileged. Respondent's decision about whether to go to the pharmacy when dealing with minor ailments depended mainly on the perceived staff's skills and direct access to medicines. Pharmacy services were little known by the public. Respondents were more willing to use autonomous prescribing than other services, with low willingness to pay out of their pocket.

Conclusions

This study shows that respondents are willing to use pharmacy services to manage minor ailments but are unaware of their existence and have low willingness to pay. Communication and uniformization of pharmacy services are needed, as well as the identification of right incentives to achieve the political goal of adequate orientation in the healthcare system when managing minor ailments.

Keywords Community pharmacy; Minor ailment; symptom management; decision making; pharmacy services

Introduction

Minor ailments are defined as “non-complicated medical conditions which can be self-diagnosed and managed, with or without the support of a healthcare professional” [1]. When people must manage minor ailments, multiple options are possible (Fig 1). They can decide to do nothing about the symptoms [2] or to use self-care by dealing with their symptoms by themselves or by asking for advice from some relatives, including or not the use of over-the-counter (OTC) medicines, but without any health professional involvement [2]. Another option is to access the healthcare system through different pathways, e.g., community pharmacy, general practitioner (GP), emergency department (ED), or other professionals according to the healthcare system [2].

Figure 1. General model of decision-making process and possible options when people must manage minor ailments [3–6]

Community pharmacies, with their expertise and accessibility, often represent an under-utilised resource in the management of minor ailments [7]. Minor ailment services [5] allow pharmacists to provide essential primary care services in a structured manner; offering information, advice, treatment and/or referral if necessary. They can deliver OTC medicines, as well as autonomous prescribing of “prescription-only medicines” according to the country’s agreements and by engaging their responsibilities [8]. Autonomous prescribing is defined as the act that occurs when “a prescriber undertakes prescribing within their scope of practice without the approval or supervision of another health professional” [9] [10].

Over the past few years, most healthcare systems have been faced with rising healthcare costs and shortages of healthcare professionals [11,12]. To address these challenges, healthcare systems encourage people with minor ailments to choose appropriate pathways, notably to avoid unnecessary ED and GP consultations [7] [13]. Extending the role of community pharmacies is one option for better resource allocation, reducing avoidable ED and GP consultations [7] [13].

However, in real life, people can find themselves confronted with numerous possible choices and may have difficulty finding their way around, depending on their individual factors and knowledge of and access to the various alternatives. Healthcare systems need to understand better how people make their choices to target communication campaigns and find the right incentives for effective orientation.

The aim of the study was to explore the criteria influencing the decision-making process when people manage minor ailments; notably the level of information of the public as well as their willingness to use and pay for pharmacy services, notably autonomous prescribing that was extended in 2019 [14]. Switzerland provides an interesting context for a case study with possible transposition to other countries/systems since people who manage minor ailments have all alternatives possible (notably pharmacy services) with a limited influence from the insurance coverage or not. Indeed, in Switzerland, people choose their mandatory health insurance contract with an annual deductible level (i.e. the amount that the insured people have to pay out of pocket for any care before the health insurance starts to cover them), going from 300 Swiss Francs (CHF) to 2’500 Swiss Francs [15] (CHF 1.00 = €1.06 or $1.16) [16] ; the insurance premium being lower with a higher deductible level. Generally, most of the 26 to 40 years old choose the 2’500 Swiss Francs deductibles (58% between 26 and 30 years old) and the older people get, the less they subscribe to this amount of deductible with 24% people between 61 and 65 years old and less than 5% for people over 80 years [17]. Indeed, people in good health will choose a high deductible level and will pay for their use of healthcare services to manage minor ailments directly out of their pocket regardless of the provider or whether the service is covered or not (e.g. pharmacy, GP, ED). In addition, people can also choose restricted insurance models, such as the requirement to consult in priority a selected GP or to contact a telemedicine service to lower their insurance premiums. The case study, therefore provides useful new insights for future studies, as the literature is currently limited almost to the UK context [2] [4] [18].

Method

A cross-sectional survey, reported in accordance with the Checklist for Reporting Of Survey Studies (CROSS) [19], was conducted from November 07, 2023 to December 08, 2023 in the French-speaking part of Switzerland.

The questionnaire S1 File was constructed using published articles and included 32 questions (plus 10 sub-questions conditional on participants' answers) divided into three parts:

- **Socio-demographic characteristics** (e.g. gender, age, education, health insurance contract modalities) [20] [21] [22];
- **General decision-making process in minor ailment management**: For three clinical scenarios that could be managed at the pharmacy (dry cough, diarrhoea, rectal bleeding) [2] [21], respondents were asked to rank preferred management options (e.g. do nothing, consult a GP, self-medication, go to the pharmacy) and assess the level of importance of criteria influencing their decision (e.g. time to deal with symptoms; health insurance model, cost, perceived symptom severity);
- **Factors influencing whether or not to choose pharmacy services for the management of minor ailments**: respondents were asked to select which of 12 criteria would prompt them to go to the pharmacy (e.g. geographical proximity, waiting time, consultation room, access to medicines, available services) [23] [24] [25] [26]; and to assess their level of knowledge, willingness to use and pay for three pharmacy services available in Switzerland: 1) autonomous prescribing, 2) prescribing via structured prescribing arrangement and 3) prescription under GP supervision were asked after the three services were defined S1 Table [27–30]. For each category of prescription, one service was chosen as an example, but they are not exhaustive of the existing pharmaceutical services.

The research team evaluated and validated the questionnaire according to the following criteria: 1) face validity (the subjective assessment of the overall impression conveyed by the questionnaire, as well as the clarity of the items and the response methods) and 2) content validity (the subjective evaluation of whether the questions align with the study objectives). The questionnaire was also piloted with four participants from the general population to assess the time required for completion and the level of comprehension. Minor changes were made based on feedback from each stage without assessing test-retest reliability due to the exploratory nature of the questionnaire. A convenience sample among the general population was recruited using two distribution methods: 1) the questionnaire was posted via social media with an invitation to transfer it to other contacts (snowball sampling method) with two recalls; 2) a flyer with a QR code heading towards the questionnaire was distributed in 50 pharmacies, four GP waiting rooms, two ED waiting rooms and a vaccination centre. Inclusion criteria were the age (≥18 years old) and to have a mandatory health insurance contract. No formal exclusion criteria were enacted, and the inclusion and identification of healthcare professionals were considered useful to enable comparison with opinions from the general population.

The data were collected and managed using the REDCap® electronic data capture tool hosted by Vanderbilt University [31]. Descriptive analyses were performed in Microsoft Excel®. Data was presented as absolute (n) / relative (%) values for categorical variables and mean/median values for quantitative ones.

Results

A total of 514 respondents completed the questionnaire. The analyses were conducted on 508 questionnaires after excluding six detected duplicates by Excel duplicate rules. Participants were mostly women aged 18 to 50 with postgraduate degrees. They were not healthcare professionals and had a restricted insurance model with a minimum annual deductible as showed in Table 1.

Table 1. Sociodemographic characteristics of respondents (n=508)

Variables	n (%)	Variables	n (%)
**Gender**		**Age range (years)**	
Female	355 (70%)	18-50	317 (63%)
Male	146 (29%)	51-64	123 (24%)
Other	4 (1%)	65-80	61 (12%)
Unknown	3 (1%)	80+	6 (1%)
		Unknown	1 (<1%)
**Education**		**Health-related profession**	
Compulsory schooling	5 (1%)	Yes	175 (34%)
Secondary vocational	93 (18%)	Pharmacy	53 (10%)
High school diploma	40 (8%)	Insurance	3 (<1%)
Professional diploma	61 (12%)	Other	115 (23%)
University, higher education	301 (59%)	Unknown	4 (<1%)
Unknown	6 (1%)	No	327 (64%)
		Unknown	6 (1%)
**Health insurance model**		**Annual deductible amount (CHF)**	
Restricted models	287 (56%)	Minimum (300)	187 (37%)
GP first (n=225)	225 (44%)	Intermediate (500 to 2000)	100 (20%)
Telemedicine (n=26)	26 (5%)	Maximum (2500)	184 (36%)
HMO* (n=22)	22 (4%)	Unknown	37 (8%)
Pharmacy/telephone helpline	4 (<1%)		
Other	8 (2%)		
Unknown	2 (<1%)		
Unrestricted model	201 (40%)		
Unknown	20 (4%)		
**Referent GP**		**Referent pharmacy**	
Yes	453 (89%)	Yes	290 (57%)
No	50 (10%)	Multiples pharmacies	92 (18%)
Unknown	5 (1%)	No (always change/ no need)	122 (24%)
		Unknown	4 (<1%)
**Perceived health status**		**Chronic illness**	
Excellent	85 (17%)	Yes	121 (24%)
Very good	171 (34%)	No	370 (73%)
Good	201 (40 %)	Multiple chronic illnesses	9 (2%)
Unsatisfying	37 (7%)	Unknown	6 (1%)
Poor	9 (2%)		
Unknown	5 (1%)		

*Health Maintenance Organisation

Preferences for the type of management were different according to the three clinical scenarios that could be managed at the pharmacy (dry cough, diarrhoea, rectal bleeding). The more serious the symptoms were perceived, the less the pharmacy and self-medication were privileged S2 Table. Depending on the symptom type, respondents chose a pharmacy to get counselling or to buy OTC S3 Table. The perceived severity of symptoms and the time to deal with symptoms were the two most critical criteria in the decision-making process (Fig 2). Cost was (very) important for only a third of respondents with different deductible amounts.

Figure 2. Criteria considered as (very) important in the decision-making process to choose the type of management preferred (Question When you answered for the three scenarios above, how did you come to your decision? What criteria did you consider?)

Respondent's decision about whether or not to go to the pharmacy when dealing with minor ailments depended mainly on the perceived staff's skills (83%, n=420) and on the direct access to medicines (81%, n=409). Health insurance coverage was important for only half of the respondents (Fig 3).

Figure 3. Criteria influencing respondents in their decision to go or not to the pharmacy (Question: What criteria do you use to decide whether or not to go to the pharmacy when you are dealing with minor ailments?)

The pharmacy services were little known by the public as well as health care professionals (excluding pharmacy-related) (Fig 4). Respondents were more willing to use autonomous prescribing than other services (Fig 5), with low willingness to pay out of their pocket. Only half and third of the respondents was willing to pay the recommended price for the services “autonomous prescribing” (15-20 Swiss francs) and “prescribing via structured prescribing

arrangement” (30-40 Swiss francs), respectively. Willingness to pay for “prescribing under GP supervision” was not questioned because the service is covered by health insurance.

Figure 4. Levels of knowledge of pharmacy services

Figure 5. Willingness to use pharmacy services

Discussion

To our knowledge, this study is the first to explore public opinion on the management of minor ailments in Switzerland, and only a few studies are available limited almost to the UK context. The subjective perceived severity of symptoms was the most important criterion in the respondents’ decision-making process, as a previous study showed [4]. The more severe the symptoms were perceived, the less self-medication and pharmacy preferred. These results corroborate previous studies stating that pharmacy and self-medication are mostly chosen when the symptoms are considered too light to consult a GP [32]. This could be due to a knowledge gap on pharmacy services and on pharmacists’ competencies as the three clinical scenarios could be managed in pharmacy (i.e. absence of red flags suggesting the need for GP consultation) [33] and more than 1,500 pharmacists have complementary training certificate in primary care anamnesis [34]. This knowledge gap has also been highlighted as a challenge in England [7]. Time to deal with symptoms was also (very) important for most of the respondents and could be linked with the perceived severity of symptoms, supposing that the more severe the symptoms were perceived, the faster resolution of symptoms was sought. The literature also shows a trade-off between time to deal with symptoms and willingness to pay additional costs [4]. Contrary to other studies [2] [4] [18] [25], the cost and the health insurance coverage did not seem to be important in the decision, probably linked to the deductible system in Switzerland since, ultimately, most people will pay for any services out of their pocket in case of minor ailments. In another

healthcare system without deductibles, the cost borne by people would be different and the financial criterion would have a different influence on the decision. We believe that limiting the influence of this important factor in our study provides interesting results that can be transposed to other contexts.

The perceived community pharmacy staff's skills and quality of the services were important criteria for respondents in deciding whether or not to go to the pharmacy. This corroborates previous results where respondents stated the need to know that pharmacists were qualified to manage minor ailments [18] [35], the need to trust the pharmacy's staff skills [32] [36] and to get a better understanding of the symptoms and their management after visiting a pharmacy [18]. Additionally, the people who visited pharmacies frequently seemed to be more willing to use minor ailment services. This could be related to good relationships with pharmacists and enhanced public awareness to their competencies [25]. Moreover, direct access to medicines was an important criterion in deciding whether or not to go to the pharmacy, and self-medication was always chosen before the pharmacy visit in each clinical scenario. This suggests that the public perceives pharmacies mainly as places where OTC medicines are bought and less as for triaging [2] [37]. Indeed, the public and health professionals (excluding pharmacy-related) were only poorly aware of pharmacy services. Better communication and visibility are needed as knowledge and awareness are crucial, i.e. because recipients cannot choose pharmacy services if they do not know them. A systematic review has highlighted these facts involving the lack of use of pharmacy services to their fullest potential [38] therefore, a communication campaign was initiated by the professional body for pharmacists Pharma Suisse [39]. In Switzerland, due to federalism and economic liberalism in the healthcare system, there is a diversity of pharmacy services with different characteristics S1 Table, driven by different stakeholders (e.g. health insurers, PharmaSuisse, health authorities). This diversity also makes it difficult for the public to understand what services are available when to use them and make the best possible choice according to the personal importance attached to each criterion. Uniformed pharmacy services to manage

minor ailments and communication supported by stakeholders would certainly enable sustaining services and grow their use and impact. For example, the NHS follows a national service strategy while maintaining some regional specificities [40] because isolated initiatives tend to be more ephemerous and struggle with sustainable funding [7]. Scotland has already used a national service for 20 years, enabling equal national service access for all eligible persons [7] [41].

Willingness to use pharmacy services are relatively high but with low willingness to pay out of their pocket**.** In Switzerland, pharmacy services are rarely covered [7] [24], and medicines costs are only covered with a prescription [42]. Moreover, the pricing of pharmacy services differs from one pharmacy to another whilst their coverage depends on the health insurance model, which could result in poor public knowledge. The willingness to pay for pharmacy services is a barrier to the use of pharmacy services (5) [43], and the Swiss population has not been used to pay for services in pharmacy, except for vaccination services [44]. The coverage of pharmacy services could be a good incentive to improve their use as it is done in the UK or Canada in minor ailments services [7,43]. Some countries, like Scotland, New Zealand and France, even go further by covering the costs of the medicines provided under minor ailments services [7]. In Switzerland, restricted insurance contracts “pharmacy model” covering pharmacy services for minor ailments services are developing in the face of rising insurance premiums. But these contracts are new and less known by the public. They compete with more popular models such as the “contact a selected GP first” and HMO models. Although a third of respondents would be willing to subscribe to such restricted insurance contracts “pharmacy model”, the impact in terms of better use of the healthcare system may not be guaranteed, as implementation of the services in community pharmacies remains low S1 Table and “pharmacy model” contracts are always mixed: people could choose other available options for minor ailments services, such as telephone helpline. Furthermore, since insureds can change insurance every year in Switzerland, it is difficult to anticipate the impact of these models on public behavior.

As for the study limitations, an accessibility bias exists as the questionnaire was only available online and in French. No preventative measures were taken to avoid multiple completions by the same individual. However, an analysis of duplicates was conducted following the collection of all questionnaires. The sample was not representative of the Swiss population as the study was exploratory. Indeed, the basic insurance contracts were overrepresented (40% vs 22%) [45]. People having restricted insurance model « Pharmacy model » (7 % vs 29 % for the 19-26 years old and 28 % for the >26 years old) [45], as well as extreme deductibles (300 Swiss francs: 37 % vs 46 % ; 2'500 Swiss francs : 36 % vs 41 %) [45] were under-represented. There was also a possible over-representation of young people [46]. A bias of desirability in favor of pharmacy services was also possible as the questionnaire was distributed in pharmacies but was minimized by having an online self-administered questionnaire. Furthermore, the QR code mostly reached persons that were already coming in pharmacies and possibly more aware of pharmacy services existence. Social media distribution helps to compensate for this bias. It is also to consider that the answers related to pharmacy services depended on how we defined them in the questionnaire. Robust quantitative findings by discrete choice experiments in Switzerland would enable a better understanding of the decision-making process and preferences for the public (e.g., possible trade-off and willingness to pay) in a context that has limited external factors impacting the decisions due to the free choice of care and the out of the pocket costs so that incentives to achieve public health objectives can be aligned with them.

Conclusion

In a system where people are free to choose any option to manage minor ailments and have mostly to pay them out of their pocket, the public is relatively willing to use pharmacy services but has little awareness and understanding of them. To achieve policy goals that encourage the population to go to the right service at the right time when they have to manage minor ailments

and to increase the contribution of pharmacies, it is necessary to develop better communication and uniformization of pharmacy services, as well as find the right incentives and strategies to guide the choice of the pharmacy. Conducting future studies in a model such as Switzerland's, which limits the influence of possible reimbursement in minor ailments services, will produce valuable knowledge for other healthcare systems.

Recommendations box

- The understanding of the decision-making process and preferences of the population in minor ailment management is crucial to reach public health objectives.
- The development of pharmacy services for managing minor ailments is an approach adopted by many healthcare systems.
- Better communication and uniformization of pharmacy services would improve their usage and impact.
- Better incentives to support the right pathways of care to minimise the burden of healthcare costs are needed.

Acknowledgements

We thank all participants for their time and their commitment.

Ethical considerations: According to the Swiss Federal Law on Research Involving Human Beings [47], no submission to the Ethics Commission for Research on Human Beings (CER-VD) [48] was required, as the data were de-identified. Tacit consent was obtained during the questionnaire's completion S1 File.

Funding statement: The survey was conducted as part of a master’s thesis in pharmacy at the University of Geneva and was financially supported by a regional organisation, the Community Pharmacist Association in Vaud region (SVPh or Société Vaudoise de Pharmacie).

Declaration of competing interest: The authors declare no conflict of interest.

References

1. Richardson M, Khouja C, Sutcliffe K, Hinds K, Brunton G, Stansfield C, Thomas J. Self care for minor ailments systematic reviews of qualitative and quantitative research. London: EPPI Centre, Social Science Research Unit, UCL Institute of Education, University College London. 2018;

2. Porteous T, Ryan M, Bond CM, Hannaford P. Preferences for self-care or professional advice for minor illness: a discrete choice experiment. British Journal of General Practice. 2006;911-7.

3. Assurance de base – Priminfo [Internet]. 2024 [cité 27 mai 2024]. Disponible sur: https://www.priminfo.admin.ch/fr/versicherungen/grundversicherung

4. Rennie L, Porteous T, Ryan M. Preferences for Managing Symptoms of Differing Severity: A Discrete Choice Experiment. Value in Health. 2012;15(8):1069-76.

5. Aly M, García-Cárdenas V, Williams K, Benrimoj SI. A review of international pharmacy-based minor ailment services and proposed service design model. Research in Social and Administrative Pharmacy. nov 2018;14(11):989-98.

6. Wyszewianski L. Access to Care: Remembering Old Lessons. Health Serv Res. déc 2002;37(6):1441-3.

7. Viegas R, Kirsten Lee K. Pharmacist-led common ailments schemes: A global intelligence report. The Netherlands: International pharmaceutical federation; 2023 p. 1-73.

8. World health organisation. The legal and regulatory framework for community pharmacies in the WHO European Region. 2019;14.

9. Pharmacy Board of Australia - Pharmacist prescribing - Position statement - 15 October 2019 [Internet]. [cité 2 nov 2024]. Disponible sur: https://www.pharmacyboard.gov.au/news/professional-practice-issues/pharmacist-prescribing-position-statement.aspx

10. Ogundipe A, Sim TF, Emmerton L. Health information communication technology evaluation frameworks for pharmacist prescribing: A systematic scoping review. Res Social Adm Pharm. févr 2023;19(2):218-34.

11. Hostettler S, Kraft E. Statistiques médicale de la FMH 2022. Bulletin des médecins suisses. 2023;104(12):24-9.

12. Bodenmann P, Graells M. Implémentation d’une intervention de Case Management dans les services d’urgence. Prim Hosp Care (fr). 2022;22(5):145-6.

13. Paudyal V, Hansford D, Cunningham S, Stewart D. Pharmacy assisted patient self care of minor ailments: A chronological review of UK health policy documents and key events 1997–2010. Health Policy. août 2011;101(3):253-9.

14. Amador-Fernández N, Botnaru I, Allemann SS, Kälin V, Berger J. Clinical relevance and implementation into daily practice of pharmacist-prescribed medication for the management of minor ailments. Front Pharmacol. 24 janv 2024;14:1256172.

15. Assurance de base – Priminfo [Internet]. [cité 27 mai 2024]. Disponible sur: https://www.priminfo.admin.ch/fr/versicherungen/grundversicherung

16. Historical Rates Tables - CHF | Xe [Internet]. [cité 6 nov 2024]. Disponible sur: https://www.xe.com/currencytables/?from=CHF&date=2024-11-05#table-section

17. Office fédéral de la santé publique OFSP, Département fédéral de l’intérieur DFI, Confédération suisse. Distribution des franchises selon la classe d’âge et le sexe Année d’exercice 2023. 2023.

18. Porteous T, Ryan M, Bond C, Watson M, Watson V. Managing Minor Ailments; The Public's Preferences for Attributes of Community Pharmacies. A Discrete Choice Experiment. Huang YC, éditeur. PLoS ONE. 2016;11(3):e0152257.

19. Sharma A, Minh Duc NT, Luu Lam Thang T, Nam NH, Ng SJ, Abbas KS, et al. A Consensus-Based Checklist for Reporting of Survey Studies (CROSS). J Gen Intern Med. oct 2021;36(10):3179-87.

20. Hermann M, Wenger V, John A. Surveillance des soins de premier recours. Zürich: Sotomo; 2022 p. 1-39.

21. Soignez-moi.ch [Internet]. 2024 [cité 3 nov 2023]. Devenir pharmacie partenaire. Disponible sur: https://www.soignez-moi.ch/pharmacies-partenaires

22. Amador-Fernández N, Matthey-de-l'Endroit J, Berger J. Factors Influencing the Implementation of a New Pharmacist Prescribing Service in Community Pharmacies. Pharmacy. 2023;11(6):173.

23. Hikaka J, Haua R, Parore N, McIntosh B, Anderson A, Pewhairangi K, et al. Designing for health equity: A mixed method study exploring community experiences and perceptions of pharmacists' role in minor ailment care. Research in Social and Administrative Pharmacy. 2023;19(4):643-52.

24. Erni P, Von Overbeck J, Reich O, Ruggli M. netCare, a new collaborative primary health care service based in Swiss community pharmacies. Research in Social and Administrative Pharmacy. 2016;12(4):622-6.

25. Bishop AC, Boyle TA, Morrison B, Barker JR, Zwicker B, Mahaffey T, et al. Public perceptions of pharmacist expanded scope of practice services in Nova Scotia. Can Pharm J. 2015;148(5):274-83.

26. De Lise M. Satisfaction et préférences du public vis-à-vis de la pharmacie dans la gestion d'une pandémie: Retour sur la COVID-19 et ses opportunités [Travail de master]. [Genève]: Unisanté; 2021.

27. Soignez-moi.ch [Internet]. 2023 [cité 26 nov 2023]. Consultation pharmacie. Disponible sur: https://www.soignez-moi.ch/consultation-pharmacie

28. Assurance de base Modèle des pharmacies – SWICA [Internet]. 2023 [cité 18 nov 2023]. Assurance de base avec première évaluation par une pharmacie ou santé24. Disponible sur: https://www.swica.ch/fr/prive/assurances/assurance-de-base/modele-des-pharmacies

29. OFSP, Office fédéral de la santé publique [Internet]. 2023 [cité 3 nov 2023]. Remise simplifiée de médicaments de la liste B. Disponible sur: https://www.bag.admin.ch/bag/fr/home/medizin-und-forschung/heilmittel/abgabe-von-arzneimitteln.html

30. netCare, Pharma Info [Internet]. 2020 [cité 26 nov 2023]. Réaliser des consultations netCare en pharmacie. Disponible sur: https://www.netcare.pharma-info.ch/?lang=fr

31. Harris PA, Taylor R, Minor BL, Elliott V, Fernandez M, O'Neal L, et al. The REDCap consortium: Building an international community of software platform partners. Journal of Biomedical Informatics. juill 2019;95:103208.

32. Mansell K, Bootsman N, Kuntz A, Taylor J. Evaluating pharmacist prescribing for minor ailments. International Journal of Pharmacy Practice. 2015;23(2):95-101.

33. Rutter P. Community pharmacy symptoms, diagnosis and treatment. 5th edition. 2020. 416 p.

34. Faits et chiffres 2023: Pharmacies suisses. Berne: Pharmasuisse; 2023 p. 1-37.

35. McKeirnan KC, Garrelts MacLean L. Pharmacist, physician, and patient opinions of pharmacist-treated minor ailments and conditions. Journal of the American Pharmacists Association. 2018;58(6):599-607.

36. Famiyeh IM, McCarthy L. Pharmacist prescribing: A scoping review about the views and experiences of patients and the public. Research in Social and Administrative Pharmacy. 2017;13(1):1-16.

37. Ansari M, Alanazi A, Moin A. Consumers' awareness, attitude and associated factors towards self-medication in Hail, Saudi Arabia. Bourgeois D, éditeur. PLoS ONE. 2020;15(4):e0232322.

38. Hindi AMK, Schafheutle EI, Jacobs S. Patient and public perspectives of community pharmacies in the United Kingdom: A systematic review. Health Expectations. avr 2018;21(2):409-28.

39. Campagne «Consultation en pharmacie» | pharmaSuisse [Internet]. [cité 2 nov 2024]. Disponible sur: https://pharmasuisse.org/fr/prestations/campagnes/consultation-en-pharmacie

40. Robinson J. Local versus national: minor ailments services across Great Britain. PJ [Internet]. 2016 [cité 28 déc 2023];297(7895). Disponible sur: https://pharmaceutical-journal.com/article/feature/local-versus-national-minor-ailments-services-across-great-britain

41. Health and social care [Internet]. 2013 [cité 7 janv 2024]. The NHS Minor Ailment Service at your local pharmacy. Disponible sur: http://www.gov.scot/publications/nhs-minor-ailment-service-local-pharmacy/

42. The compulsory health insurance system: Guide [Internet]. Berne: Federal Department of Public Health FOPH, Swiss Confederation; 2024 févr. Disponible sur: https://www.bag.admin.ch/dam/bag/en/dokumente/kuv-aufsicht/krankenversicherung/sie-fragen-wir-antworten-oblig-kv.pdf.download.pdf/broschuere-sie-fragen-wir-antworten-e.pdf

43. Taylor J, Joubert R. Pharmacist-led minor ailment programs: a Canadian perspective. IJGM. 2016;Volume 9:291-302.

44. Leuthold C, Bugnon O, Berger J. The Role of Community Pharmacists in Travel Health and Vaccination in Switzerland. Pharmacy. 29 nov 2018;6(4):125.

45. Grize S. Statistique de l’assurance-maladie obligatoire Edition 2021. Berne: OFSP, Office fédéral de la santé publique; 2023. Report No.: 6.

46. OFS, Office fédéral de la statistique [Internet]. [cité 26 déc 2023]. Age. Disponible sur: https://www.bfs.admin.ch/bfs/fr/home/statistiken/bevoelkerung/stand-entwicklung/alter.html

47. Office fédéral de la santé publique (OFSP). Federal Act on Research involving human beings (Human Research Act, HRA) [Internet]. 2011 [cité 21 mai 2024]. Disponible sur: Office Fédéral de la Santé Publique (OFSP). Loi fédérale relative à la recherche sur l'être humain (LRH) 2011. Available from: fedlex-dta-admin-ch-eli-cc-2013-617-20200101-fr-pdf-a.pdf [Access date: 19.12.23]

48. CER-VD [Internet]. 2024 [cité 21 mai 2024]. Comission cantonale d’éthique de la recherche sur l’être humain (CER-VD). Disponible sur: https://www.cer-vd.ch

Supporting information

S1 Table. Examples of pharmacy services to manage minor ailments available in Switzerland

S1 File. Questionnaire "Exploration of the opinions in the management of minor ailments"

S2 Table. Orientation decision based on perceived severity of symptoms

S3 Table. Results for the question: "*Indicate the minor ailments that would make you visit a pharmacy (yes I go to the pharmacy for a consultation or a counselling, yes I go to the pharmacy to buy some medicines without prescriptions, no I don't go to the pharmacy if I have these symptoms, unapplicable, I don't know, I don't want to answer*"

Click here to access/download;Figure;Fig 1.png

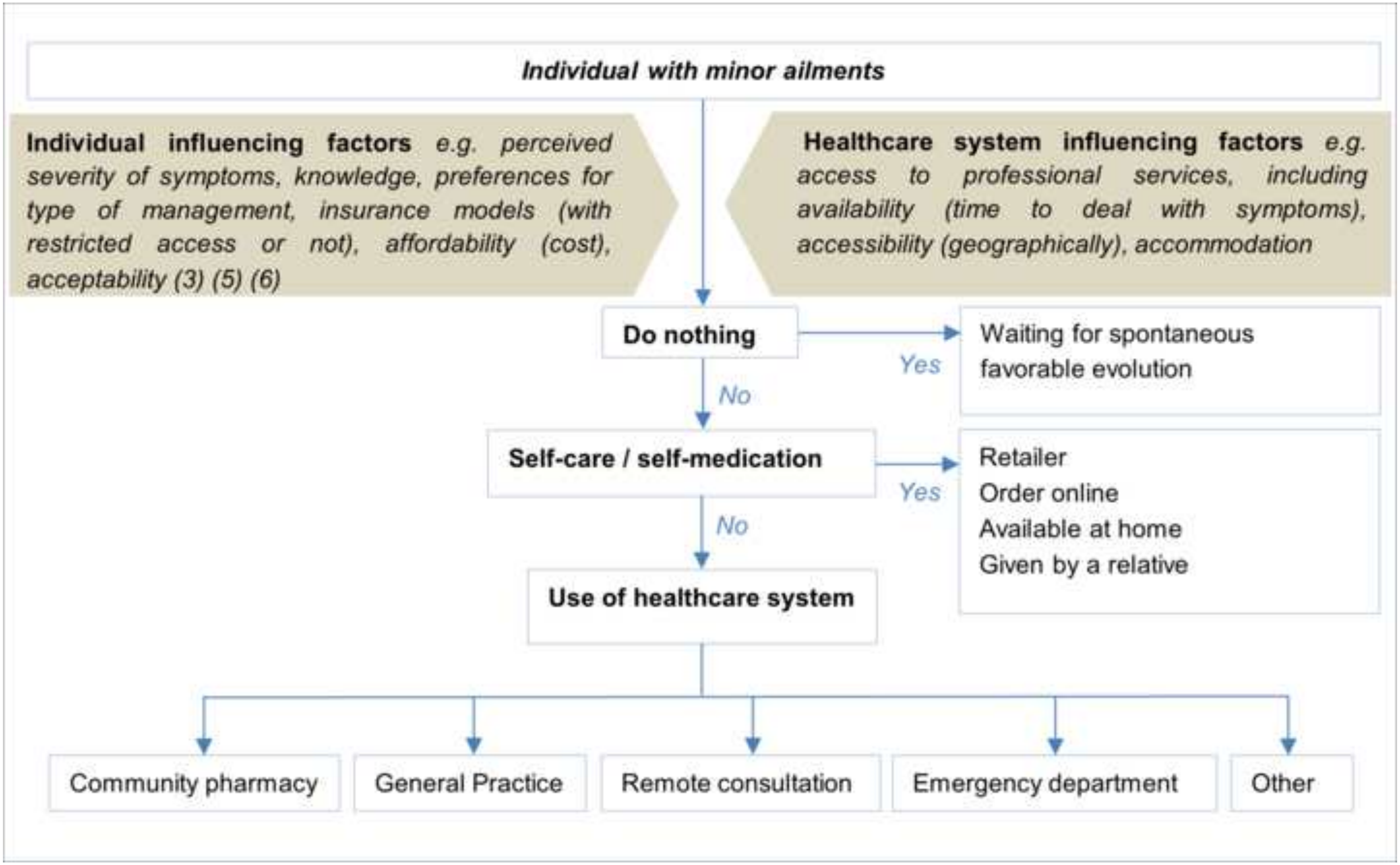

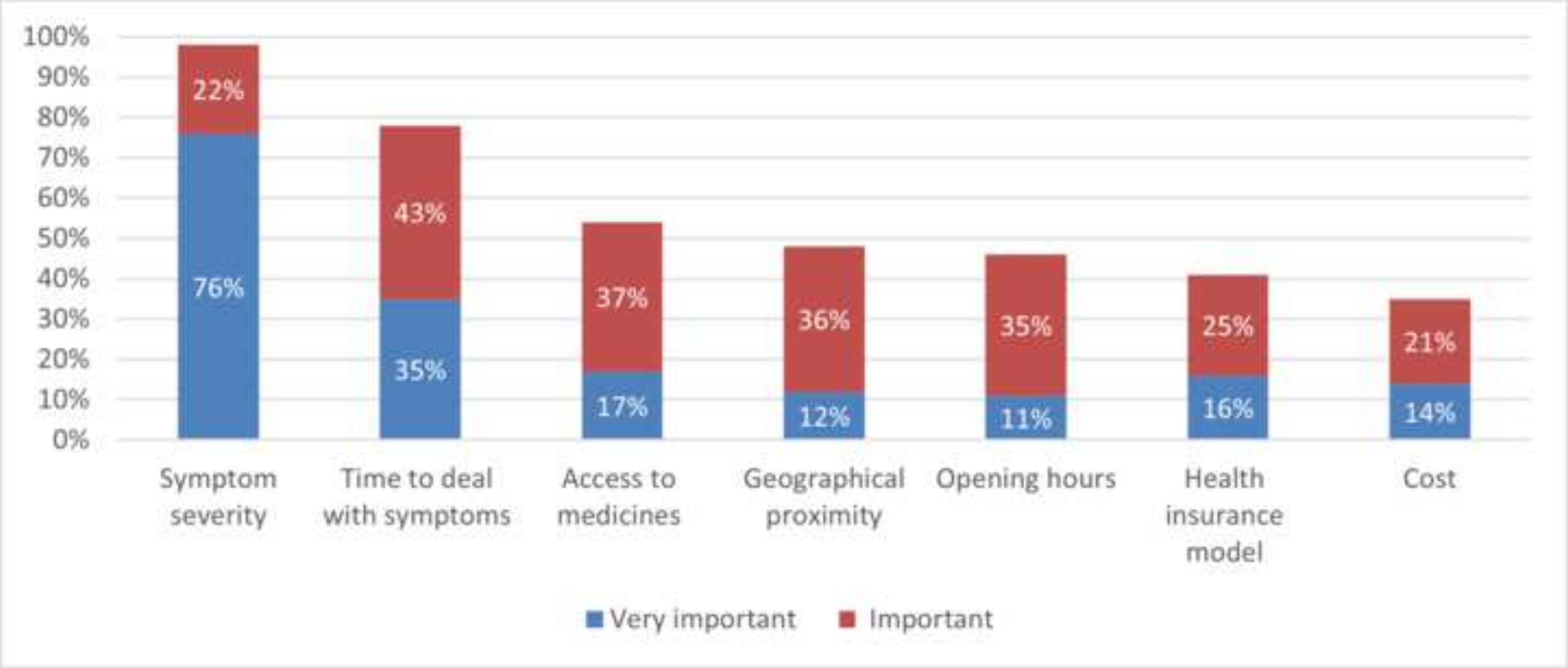
100%
90%
80%
70%
60%
50%
40%
30%
20%
10%
0%
22%
76%
43%
35%
37%
17%
36%
12%
35%
11%
25%
16%
21%
14%
Symptom severity
Time to deal with symptoms
Access to medicines
Geographical proximity
Opening hours
Health insurance model
Cost
Very important
Important

Click here to access/download;Figure;Fig 3.png

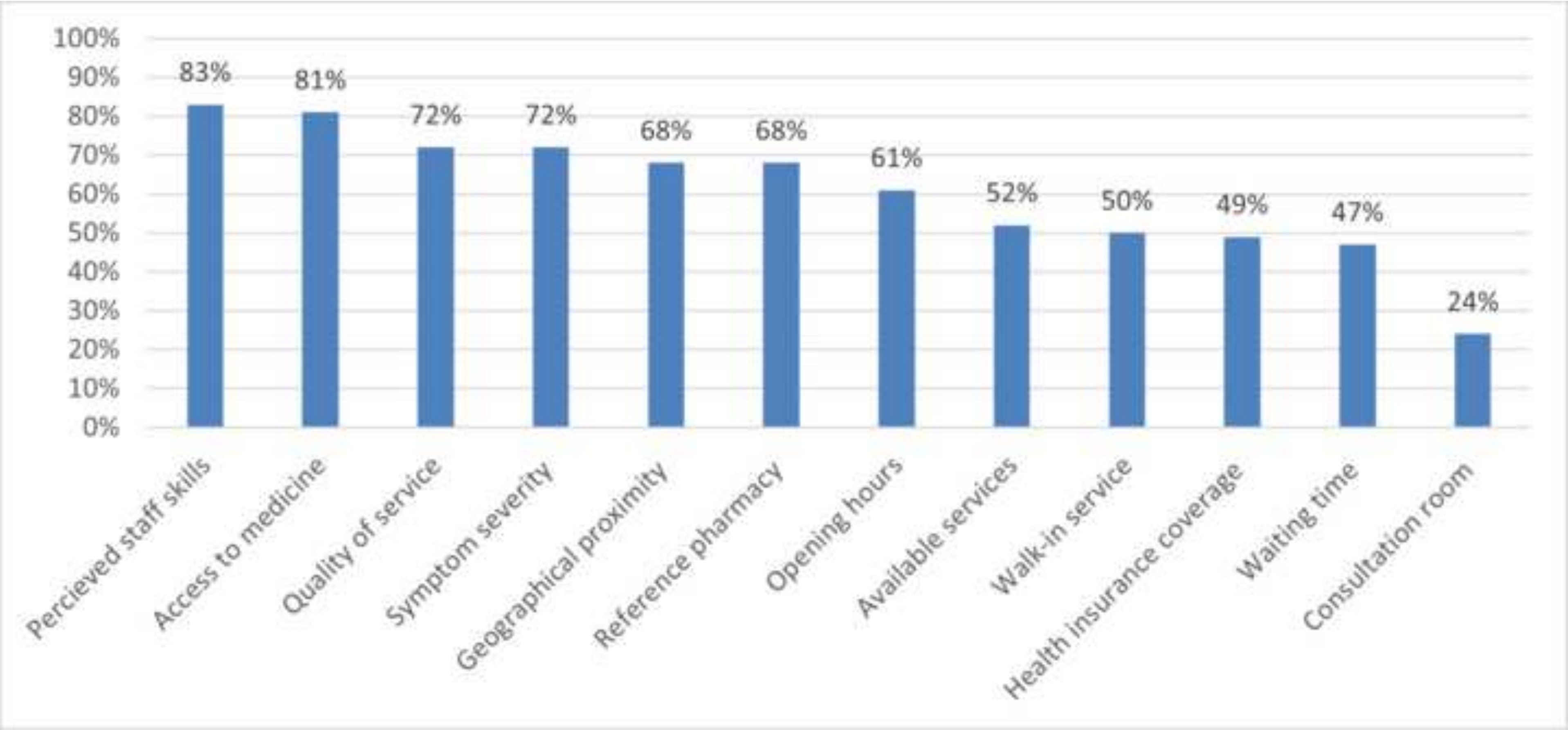

Click here to access/download;Figure;Fig 4.png

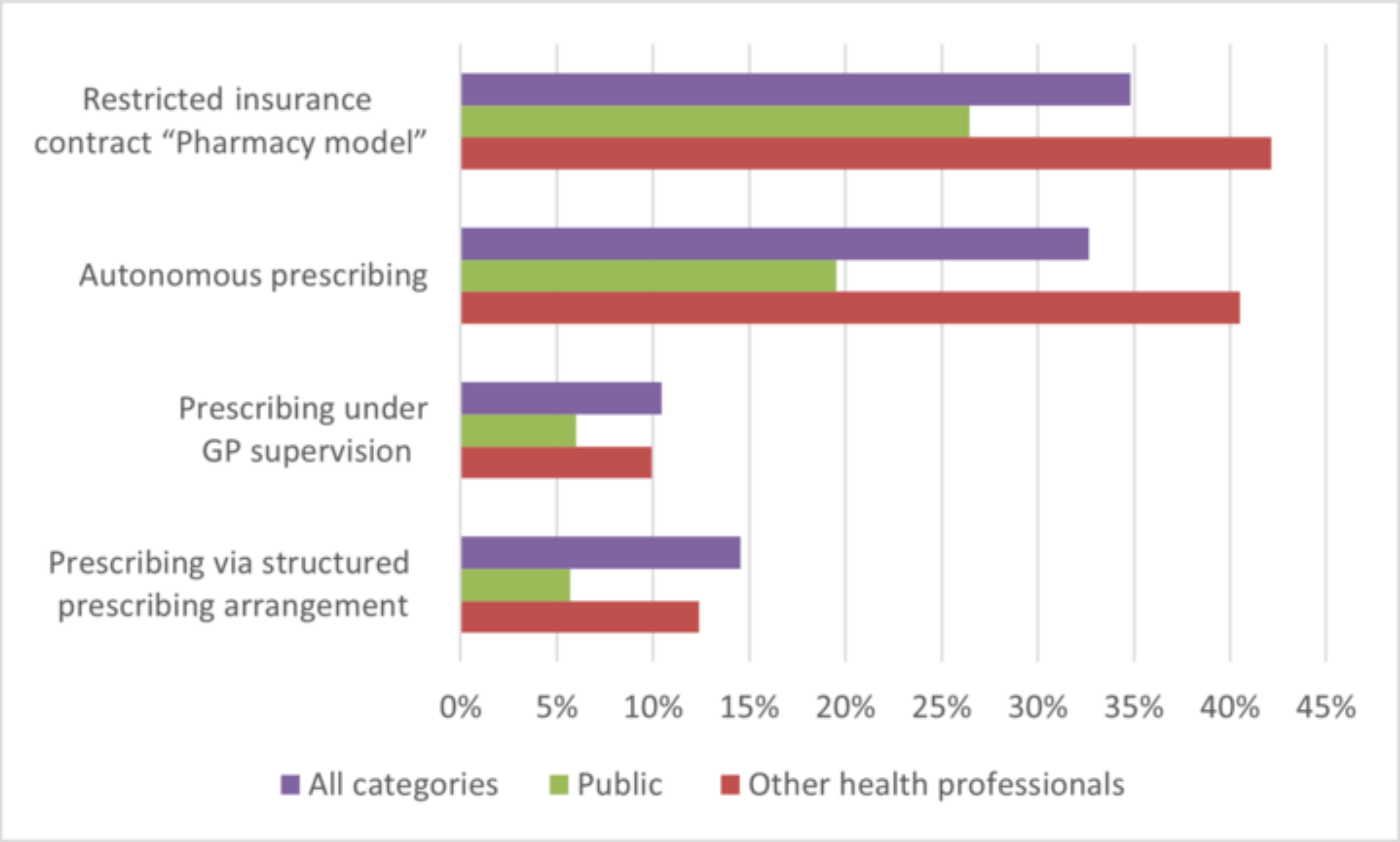

Click here to access/download;Figure;Fig 5.png

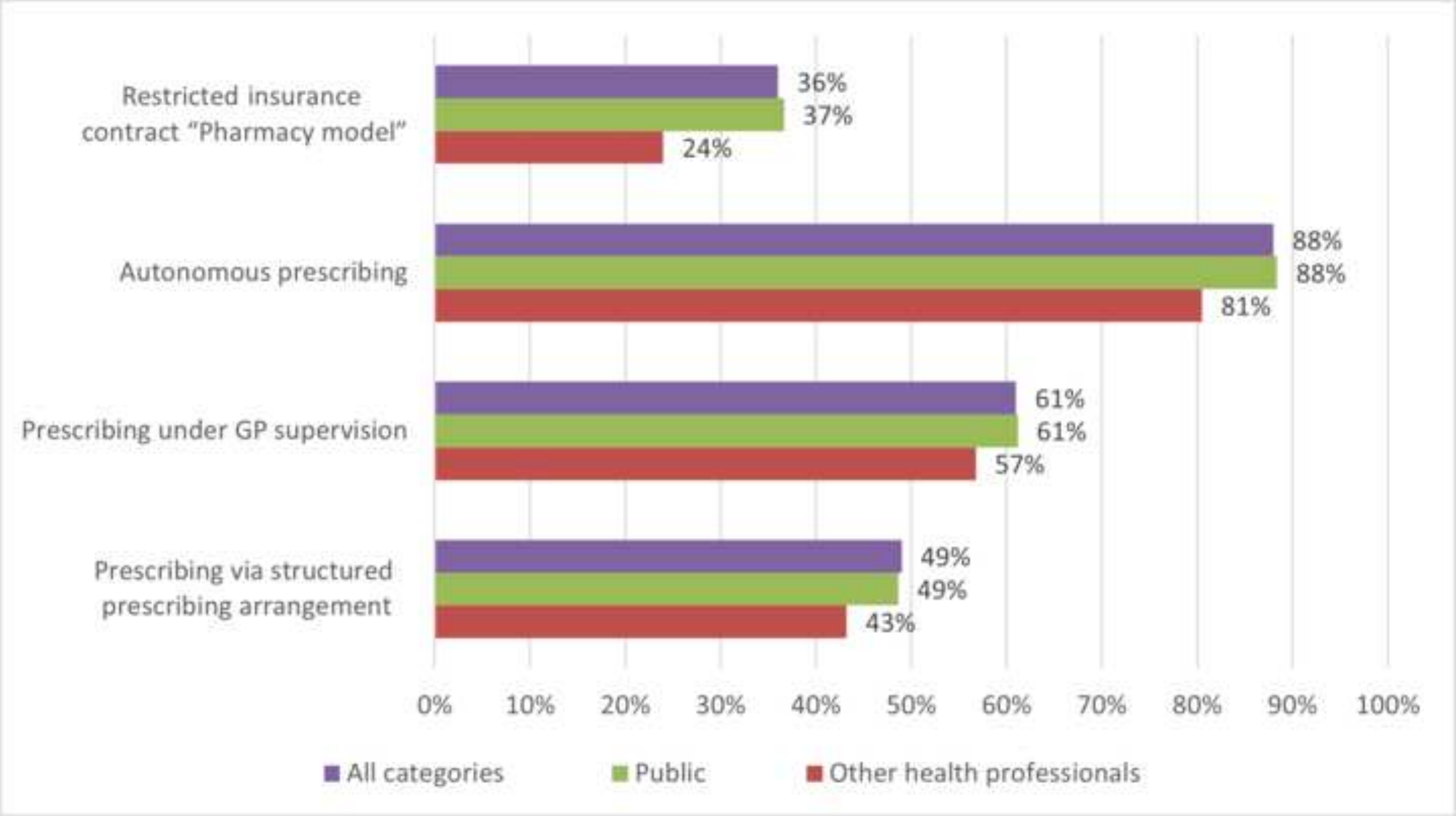

Click here to access/download
Supporting Information
S1 File.pdf

Click here to access/download
Supporting Information
S1 Table.docx

Click here to access/download
Supporting Information
S2 Table.docx

Click here to access/download
Supporting Information
S2 Table.pdf

Click here to access/download
Supporting Information
S3 Table.docx

Click here to access/download
Supporting Information
S3 Table.pdf
