# Supplementary material for: Managing minor ailments and pharmacy services: How do people make their decisions?
Source: PLoS One. 2025 Aug 26;20(8):e0330706. doi: 10.1371/journal.pone.0330706 (PMC12380283; doi:10.1371/journal.pone.0330706)
Supplement: Attachment — Submitted filename: 25.04.15 Review Minor ailments PONE-D-25-05729 .docx [file pone.0330706.s005.docx]

It's interesting to learn about this through a case study conducted in a country other than the UK, Australia, or Canada, where most studies on community pharmacy and minor ailment services are conducted. I also think the findings of this paper are applicable to other countries, since although people are willing to use pharmacy services to manage minor ailments, they are unaware of their existence and have a low willingness to pay.

In the Methods section (line 122), the authors state that the questionnaire used was evaluated and “validated” by the research team. I don't think the word "validated" is correct, since in my opinion, validating a questionnaire requires assessing both reliability (something the authors do when evaluating Face and Content validity) and feasibility (alpha Cronbach or any other test). Therefore, I would modify the text ("The research team evaluated and “assessed” the questionnaire according to the...").

In Results, I think the text should be clarified a bit. The authors say (lines 144-145): “They were not healthcare professionals and had a restricted insurance model with a minimum annual deductible, as shown in Table 1”. In reality, those who had "restricted models" were 56%, which, although it is the majority, is a percentage close to 50%. In my opinion, it does not justify the generalization used since it begins with "They were." I think it would be better to start with “the majority being non-healthcare professionals”.

It is interesting to see that, although as described in table S2, the more serious the symptoms were, the less self-medication and pharmacy were preferred, in Figure 3, “perceived severity” is the fourth cause for going to the pharmacy. This should surely be discussed in the Discussion because at first glance it does not make sense. In fact, the discussion says (line 183) that “The more severe the symptoms were perceived, the less self-medication and pharmacy preferred”, but it does not add that the severity of symptoms was the fourth cause for going to the pharmacy.

(Line 219) In line 219 when the authors state that “there is a diversity of pharmacy services with different characteristics” I would clarify that they refer to “different services to manage minor ailments” to avoid confusion with other different services such as Medication review, adherence, reconciliation and so on.

Regarding REFERENCES, authors should always follow the same criteria when describing the journal: either use the full name, as in Ref#2, or use the abbreviated name, as they do in Ref#14:

Ref#1. A semicolon should follow London, before the year.

Ref#2. The volume is missing: BJGP 2006; 56(533):911-7

Ref#5. Delete “Nov”

Ref#6. Delete “Dec”

Ref#8. They should include a link to the website.

Ref#10. Delete “Fevr”

Ref#13. Delete “Aut”

Ref#14. Delete “24 Janv”

Ref#17. A link to the website should be included.

Ref#19. Delete “Oct”

Ref#31. Delete “Jull”

Ref#34. I would reference this as: Rutter P. Community pharmacy symptoms, diagnosis and treatment. 5th edition. London: Elsevier ed.; 2020. 416 p.

Ref#34. A link to the website should be included.

Ref#43. Or, Int J Gen Med, or include the full name of the journal. Volume (2016;9:291-302).

Ref#44. Basel is missing from the journal name, and delete "Nov 29": Pharmacy (Basel). 2018:6(4):125.

Ref#45. A link to the website should be included.
