# Supplementary material for: Managing minor ailments and pharmacy services: How do people make their decisions?
Source: PLoS One. 2025 Aug 26;20(8):e0330706. doi: 10.1371/journal.pone.0330706 (PMC12380283; doi:10.1371/journal.pone.0330706)
Supplement: Attachment — Submitted filename: Response to reviewers.docx [file pone.0330706.s008.docx]

**Journal Requirements:**

Additionally, please revise the percentages reported in **Table 1**. Some values listed as 1% are actually **less than 1%** (e.g., gender), and the current presentation results in a total exceeding 100%. Thanks for the comment. *Percentages in the table have been rounded off and values below 1 noted as “<1%” to correspond as closely as possible to 100%.*

1. When submitting your revision, we need you to address these additional requirements.

Please ensure that your manuscript meets PLOS ONE's style requirements, including those for file naming. The PLOS ONE style templates can be found at

https://journals.plos.org/plosone/s/file?id=wjVg/PLOSOne_formatting_sample_main_body.pdf and https://journals.plos.org/plosone/s/file?id=ba62/PLOSOne_formatting_sample_title_authors_affiliations.pdf

2. Thank you for stating in your Funding Statement:

All the master’s thesis in pharmacy at the University of Geneva and conducted at Unisanté are financially supported by a regional organisation, the Community Pharmacist Association in Vaud region (SVPh or Société Vaudoise de Pharmacie).

Please provide an amended statement that declares *all* the funding or sources of support (whether external or internal to your organization) received during this study, as detailed online in our guide for authors at <http://journals.plos.org/plosone/s/submit-now>.  Please also include the statement “There was no additional external funding received for this study.” in your updated Funding Statement.

Please include your amended Funding Statement within your cover letter. We will change the online submission form on your behalf.

*The funding statement has been changed (see below). The organisation does not financially support author individually and no grant number is associated to the research as the support is general. Funding statement : “**The survey was carried out as part of a master's thesis in pharmacy at the University of Geneva. Unisanté receives global financial support for the supervision of all master's theses in pharmacy at the University of Geneva from a regional organization, the Société Vaudoise de Pharmacie (SVPh; https://www.svph.ch/). The funder plays no role in the research resulting from these theses****.*** *There was no additional external funding received for this study.****”***

3. We note that you have indicated that there are restrictions to data sharing for this study. For studies involving human research participant data or other sensitive data, we encourage authors to share de-identified or anonymized data. However, when data cannot be publicly shared for ethical reasons, we allow authors to make their data sets available upon request. For information on unacceptable data access restrictions, please see http://journals.plos.org/plosone/s/data-availability#loc-unacceptable-data-access-restrictions.

Before we proceed with your manuscript, please address the following prompts:

a) If there are ethical or legal restrictions on sharing a de-identified data set, please explain them in detail (e.g., data contain potentially identifying or sensitive patient information, data are owned by a third-party organization, etc.) and who has imposed them (e.g., a Research Ethics Committee or Institutional Review Board, etc.). Please also provide contact information for a data access committee, ethics committee, or other institutional body to which data requests may be sent.

b) If there are no restrictions, please upload the minimal anonymized data set necessary to replicate your study findings to a stable, public repository and provide us with the relevant URLs, DOIs, or accession numbers. Please see http://www.bmj.com/content/340/bmj.c181.long for guidelines on how to de-identify and prepare clinical data for publication. For a list of recommended repositories, please see https://journals.plos.org/plosone/s/recommended-repositories. You also have the option of uploading the data as Supporting Information files, but we would recommend depositing data directly to a data repository if possible.

Please update your Data Availability statement in the submission form accordingly.

*Thanks to this comment. After checking with our colleague at the institutional data repository, the dataset is now available in open access. Five columns with text comments have been removed for a de-identifying dataset.*

*Access request can be made by research teams at :* [*https://doi.org/10.16909/dataset/56*](https://doi.org/10.16909/dataset/56)

*Survey ID number :* *10.16909-dataset-56*

4. Your ethics statement should only appear in the Methods section of your manuscript. If your ethics statement is written in any section besides the Methods, please move it to the Methods section and delete it from any other section. Please ensure that your ethics statement is included in your manuscript, as the ethics statement entered into the online submission form will not be published alongside your manuscript. *Ethics statement has been moved to Materials and methods section (page 7 lines 141-146)*

5. We note that this data set consists of interview transcripts. Can you please confirm that all participants gave consent for interview transcript to be published? *No, data only include response to questionnaire S1 file.*

If they DID provide consent for these transcripts to be published, please also confirm that the transcripts do not contain any potentially identifying information (or let us know if the participants consented to having their personal details published and made publicly available). We consider the following details to be identifying information:

- Names, nicknames, and initials

- Age more specific than round numbers

- GPS coordinates, physical addresses, IP addresses, email addresses

- Information in small sample sizes (e.g. 40 students from X class in X year at X university)

- Specific dates (e.g. visit dates, interview dates)

- ID numbers

Or, if the participants DID NOT provide consent for these transcripts to be published:

- Provide a de-identified version of the data or excerpts of interview responses

- Provide information regarding how these transcripts can be accessed by researchers who meet the criteria for access to confidential data, including:

a) the grounds for restriction

b) the name of the ethics committee, Institutional Review Board, or third-party organization that is imposing sharing restrictions on the data

c) a non-author, institutional point of contact that is able to field data access queries, in the interest of maintaining long-term data accessibility.

d) Any relevant data set names, URLs, DOIs, etc. that an independent researcher would need in order to request your minimal data set.

For further information on sharing data that contains sensitive participant information, please see: <https://journals.plos.org/plosone/s/data-availability#loc-human-research-participant-data-and-other-sensitive-data>

If there are ethical, legal, or third-party restrictions upon your dataset, you must provide all of the following details (<https://journals.plos.org/plosone/s/data-availability#loc-acceptable-data-access-restrictions>):

a) A complete description of the dataset

b) The nature of the restrictions upon the data (ethical, legal, or owned by a third party) and the reasoning behind them

c) The full name of the body imposing the restrictions upon your dataset (ethics committee, institution, data access committee, etc)

d) If the data are owned by a third party, confirmation of whether the authors received any special privileges in accessing the data that other researchers would not have

e) Direct, non-author contact information (preferably email) for the body imposing the restrictions upon the data, to which data access requests can be sent

6. Please review your reference list to ensure that it is complete and correct. If you have cited papers that have been retracted, please include the rationale for doing so in the manuscript text, or remove these references and replace them with relevant current references. Any changes to the reference list should be mentioned in the rebuttal letter that accompanies your revised manuscript. If you need to cite a retracted article, indicate the article’s retracted status in the References list and also include a citation and full reference for the retraction notice. *The reference list has been only modified to Vancouver format according to author instructions.*

**Comments to the Author**

Reviewer's Responses to Questions

1. Is the manuscript technically sound, and do the data support the conclusions?

The manuscript must describe a technically sound piece of scientific research with data that supports the conclusions. Experiments must have been conducted rigorously, with appropriate controls, replication, and sample sizes. The conclusions must be drawn appropriately based on the data presented.

Reviewer #1: Yes

Reviewer #2: Yes

2. Has the statistical analysis been performed appropriately and rigorously?

Reviewer #1: Yes

Reviewer #2: Yes

3. Have the authors made all data underlying the findings in their manuscript fully available?

The [PLOS Data policy](http://track.editorialmanager.com/CL0/http:%2F%2Fwww.plosone.org%2Fstatic%2Fpolicies.action%23sharing/1/010f01969fcb4f3c-554a9829-9344-41c3-bba0-06e99a2dbe45-000000/xtIRlHWO2yBPwEp1d6B8fc0iSanuo0SWvBExdV2wV7g=209) requires authors to make all data underlying the findings described in their manuscript fully available without restriction, with rare exception (please refer to the Data Availability Statement in the manuscript PDF file). The data should be provided as part of the manuscript or its supporting information, or deposited to a public repository. For example, in addition to summary statistics, the data points behind means, medians and variance measures should be available. If there are restrictions on publicly sharing data—e.g. participant privacy or use of data from a third party—those must be specified.

Reviewer #1: Yes

Reviewer #2: Yes

4. Is the manuscript presented in an intelligible fashion and written in standard English?

PLOS ONE does not copyedit accepted manuscripts, so the language in submitted articles must be clear, correct, and unambiguous. Any typographical or grammatical errors should be corrected at revision, so please note any specific errors here.

Reviewer #1: Yes

Reviewer #2: Yes

**5. Review Comments to the Author**
*Please use the space provided to explain your answers to the questions above. You may also include additional comments for the author, including concerns about dual publication, research ethics, or publication ethics. (Please upload your review as an attachment if it exceeds 20,000 characters)*

**Reviewer #1:** It's interesting to learn about this through a case study conducted in a country other than the UK, Australia, or Canada, where most studies on community pharmacy and minor ailment services are conducted. I also think the findings of this paper are applicable to other countries, since although people are willing to use pharmacy services to manage minor ailments, they are unaware of their existence and have a low willingness to pay. *Thanks for your comment, we also believe that publishing illustrations outside the UK, Australia and Canada is important for international literature. Linking to a comment from reviewer 2, we have added this to the strengths of the study: “This protocol is the first of its kind in Switzerland and provides an initial insight into people’s opinions and the decision-making process regarding the management of minor ailments in this population. The interest of this study is to examine these decision-making processes in a different context from other countries than the UK, Australia or Canada, where most studies on community pharmacy and minor ailment services are conducted. In Switzerland, due to the deductible system, most healthy adults using a health service to treat a minor ailment would pay for the service out of their own pocket, regardless of the provider. We believe that limiting the influence of the cost factor provides interesting results that can be transposed to other contexts.” (discussion section, page14 lines 266-273)*

In the Methods section (line 122), the authors state that the questionnaire used was evaluated and “validated” by the research team. I don't think the word "validated" is correct, since in my opinion, validating a questionnaire requires assessing both reliability (something the authors do when evaluating Face and Content validity) and feasibility (alpha Cronbach or any other test). Therefore, I would modify the text ("The research team evaluated and “assessed” the questionnaire according to the..."). *The sentence has been modified as suggested (section materials and methods page 7 lines 125-128).*

In Results, I think the text should be clarified a bit. The authors say (lines 144-145): “They were not healthcare professionals and had a restricted insurance model with a minimum annual deductible, as shown in Table 1”. In reality, those who had "restricted models" were 56%, which, although it is the majority, is a percentage close to 50%. In my opinion, it does not justify the generalization used since it begins with "They were." I think it would be better to start with “the majority being non-healthcare professionals”. *You are perfectly right. The sentence has been modified as suggested: “The majority being not-healthcare professionals females with postgraduate degrees in good health and with a restricted insurance model as showed in Table 1.” (section results, page 8 lines 148-150).*

It is interesting to see that, although as described in table S2, the more serious the symptoms were, the less self-medication and pharmacy were preferred, in Figure 3, “perceived severity” is the fourth cause for going to the pharmacy. This should surely be discussed in the Discussion because at first glance it does not make sense. In fact, the discussion says (line 183) that “The more severe the symptoms were perceived, the less self-medication and pharmacy preferred”, but it does not add that the severity of symptoms was the fourth cause for going to the pharmacy.  *We do not interpret these two results as ambivalent. Indeed,* *Figure 3 highlights the fact that “****perceived*** *severity of symptoms” is an important criterion* *in determining whether or not to go to the pharmacy. However, this result does not directly indicate whether people are more likely to visit a pharmacy if their symptoms are more or less severe, since the question is not formulated in this way. What's interesting to note here is that this criterion is the first in the ranking not to be linked to the characteristics of a particular pharmacy. It is the elements presented in S2 that allow us to interpret that the more severe the symptoms, the less likely people are to go to the pharmacy. We added the precision on the discussion section (page 11 lines 199-202): “Moreover, “perceived severity of symptoms” is an important criterion for going to the pharmacy (Fig 3) and the more severe the symptoms were perceived, the less self-medication and pharmacy preferred (S2 Table).”*

(Line 219) In line 219 when the authors state that “there is a diversity of pharmacy services with different characteristics” I would clarify that they refer to “different services to manage minor ailments” to avoid *confusion with other different services such as Medication review, adherence, reconciliation and so on. Thanks, we have added the adding as suggested to precise (section discussion page 12 lines 232-235)*

Regarding REFERENCES, authors should always follow the same criteria when describing the journal: either use the full name, as in Ref#2, or use the abbreviated name, as they do in Ref#14:
Ref#1. A semicolon should follow London, before the year.
Ref#2. The volume is missing: BJGP 2006; 56(533):911-7
Ref#5. Delete “Nov”
Ref#6. Delete “Dec”
Ref#8. They should include a link to the website.
Ref#10. Delete “Fevr”
Ref#13. Delete “Aut”
Ref#14. Delete “24 Janv”
Ref#17. A link to the website should be included.
Ref#19. Delete “Oct”
Ref#31. Delete “Jull”
Ref#34. I would reference this as: Rutter P. Community pharmacy symptoms, diagnosis and treatment. 5th edition. London: Elsevier ed.; 2020. 416 p.
Ref#34. A link to the website should be included.
Ref#43. Or, Int J Gen Med, or include the full name of the journal. Volume (2016;9:291-302).
Ref#44. Basel is missing from the journal name, and delete "Nov 29": Pharmacy (Basel). 2018:6(4):125.
Ref#45. A link to the website should be included.

**Reviewer #2:** [*The page numbers below refer to the submitted pdf, the reviewer's comments have been transcribed in this document to respond to them.]*

**Abstract**

Page 10 : Please describe major results in numbers and percentages.

Page 11 : Please add more details about the significance of this.

*Thanks for these suggestions to improve this section. We modified the abstract in adding major numbers and percentages and improve the significance of the findings. We also adopt a more active forms to improve it. New abstract :* ***Background*** *Healthcare systems face challenges such as rising costs and workforce shortages. Optimal resource allocation is needed, notably in the management of minor ailments. Community pharmacy services (e.g. minor ailment schemes) remain underused by the population. This study explores the decision-making process when people are managing minor ailments: What influences their choices among the management options? What is their level of information and willingness to use and pay for pharmacy services?* ***Methods*** *A cross-sectional online survey was conducted using a self-administered questionnaire (convenience sampling) from 07.11.2023 to 08.12.2023 in Switzerland. The survey explored general decision-making process in minor ailment management through three clinical scenarios, factors influencing the choice of pharmacy services, as well as public knowledge of three available pharmacy services and their willingness to use and pay for them.* ***Results*** *A total of 508 valid responses were analysed (99.8%). Perceived severity of symptoms (98%, n=495) and time to deal with symptoms (78%, n=396) were the most important criteria in making the management decision. The more serious the symptoms were perceived, the less the pharmacy and self-medication were privileged. Choosing pharmacy depended mainly on the perceived staff's skills (83%, n=420) and direct access to medicines (81%, n=409). Public awareness of pharmacy services was low. Respondents were more willing to use autonomous prescribing than other services, with low willingness to pay out of their pocket.* ***Conclusions*** *There is a critical gap between the potential demand for minor ailment services in pharmacy and the public’s awareness and valuation of them. Actual uptakes could be improved by better communication and uniformization of pharmacy services, as well as the identification of right incentives to achieve the political goal of adequate orientation in the healthcare.”*

**Introduction**

Page 12: Starting your introduction directly with a term definition is not a good start. please add paragraph related focus on your study title then you can add the "Minor Ailments" definition. *Thanks for this comment. We have added a sentence to precise the rational of the problem before the definition (page 4 lines 47-49). “While people's choices when managing minor ailments have a direct impact on healthcare systems (availability of professionals, waiting times, cost), their decision-making process is little studied.”*

Page 12: I am not sure about having figure in the introduction as this would be more suitable in thesis (academic) writing and not suitable in writing for publication. *The figure has been moved in the materials and method section with the following sentence (page 6 lines 100-102): “The questionnaire (S1 File) was constructed from a general model of decision-making and possible options when people must manage minor ailments, created on the basis of published articles (Fig 1).”*

**Method**

Page 14: rewrite these three parts in a paragraph style. Again this is more thesis (academic) writing, not a good way of writing for "Publication". Please rewrite. *Thanks, we have modified in a paragraph style as suggested (page 6 lines 107-123).*

Page 15: Please add details about the content validation process. what you have said about content validity "" is not what content validity is all about. *Thank you for the comment in agreement with reviewer 1. Indeed, we have only assessed face and content validity, but not the feasibility (e.g. alpha Cronbach or any other test). We have changed the paragraph to reflect this comment. (page 7 lines 124-131)*

Page 15: Add numbers and percentages. *All numbers and percentages are shown in Table 1. To facilitate the reading, we just propose to keep the sentence as it stands to illustrate the typical profile of respondents (the sentence has been modified according to the comments of reviewer 1) (page 8 lines 148-150)*

**Discussion**

Page 18: add a small paragraph describing the significance of your study related to your study findings, then add "to our knowledge ........" *We have added a paragraph at the beginning of the discussion to synthesize the main findings: “The present study explored the decision-making process when people are managing minor ailments and their willingness to use and pay for pharmacy services available in Switzerland. The study showed that perceived severity of symptoms (98%, n=495) and time to deal with symptoms (78%, n=396) were the most important criteria in making the management decision. They are willing to go to pharmacy, mainly driven by the perceived staff’s skills (183%, n=420) and direct access to medicines (81%, n=409). Public awareness of pharmacy services was low. Respondents were more willing to use autonomous prescribing than other services, with low willingness to pay out of their pocket.” (page 10 line 186 – page 11 line 193)*

Page 21: does your study have any strengths? if yes, please add them here.

*Yes ! we have added the strengths of our study before the limits; and we have also created subheadings in this section for greater clarity. “****Strengths and limits*** *This protocol is the first of its kind in Switzerland and provides an initial insight into people’s opinions and the decision-making process regarding the management of minor ailments in this population. The interest of this study is to examine these decision-making processes in a different context from other countries than the UK, Australia or Canada, where most studies on community pharmacy and minor ailment services are conducted. In Switzerland, due to the deductible system, most healthy adults using a health service to treat a minor ailment would pay for the service out of their own pocket, regardless of the provider. We believe that limiting the influence of the cost factor provides interesting results that can be transposed to other contexts.” (page 13 line 264 – page 14 line 272).*

Page 21: does your study findings have any impacts to practice? is/are there any key messages you want to send to healthcare professionals of policymakers. Please add them clearly in a paragraph (the last paragraph of your discussion). *The key messages for researchers, professionals and policymakers have been added in the last paragraph of our discussion (replacing the “recommendations box”)**. “Understanding how individuals make decisions and express preferences in managing minor ailments is essential for achieving public health goals. For researchers, this paper provides a foundation for designing studies that better reflect real-world behaviors and inform evidence-based interventions. In Switzerland, pharmacists and policymakers should do more to communicate the fact that a health service to treat a minor ailment is in most cases paid for out-of-pocket by patients, and that treatment in a pharmacy costs less than other alternatives while limiting waiting times and providing direct access to a drug if necessary. Finally, pharmacies should effectively implement the services and successfully capture potential demand.” (page 15 lines 292-299)*

**Conclusion**

Page 21: rewrite the whole conclusion section.

direction for the authors:

1. start with your number one major findings.,

2. then add future research and reconsecrations.

*Thanks for this suggestion. As major findings have been already added in the discussion and abstract, we assume not rewrite here, following the actual authors guideline of Plos One. However, we have totally rewrite the conclusion in the direction that seemed relevant to us following your comments: “The paper shows a critical gap between the potential demand for minor ailment services in pharmacy and the public’s awareness and valuation of them in Switzerland, but which is potentially just as true in many countries. Actual uptakes could be improved by better communication and design/uniformization of pharmacy services, as well as find the right incentives and strategies to guide the choice of the pharmacy. Future research needs to be conducted nationally to find the effective combination of multi-level and multi-dimensional implementation strategies that will achieve public health goals.” (page 15 lines 302-308).*

**Recommendations box**

Page 22: not sure about the validity of having this recommendation box (i.e.., this is suitable for academic writing, but not suitable way of writing for publication). Please check with author guidelines. *No specific guidelines about this section. See the answer above. Instead of this box, which has been deleted, recommendations have been added and rewritten in the last paragraph of the discussion.*
